# Cases at the Medical Dispensary, Medical Department, University of Buffalo

**Published:** 1847-05

**Authors:** 


ART. II.—*Cases at the Medical Dispensary, Medical Department, University of Buffalo.*

The following are selected from the cases presenting themselves at the College Medical Dispensary, of which notes have been preserved. Of a considerable number, which occurred during the early part of the session, no records were made, and of those in which minutes were kept, a complete report would occupy too much space. The report will probably be continued.

*March 2d.—Cephalalgia.*—S., aet. 30. Complains of acute lancinating pain in the head, affecting the frontal and occipital portions. The pain is not constant. but occasional, recurring, more or less, every week ; occurs suddenly and, after continuing a time, goes off as suddenly. As he is a labourer, dependent upon his daily exertions for support, it is a serious inconvenience. Has tried various remedies without relief. Among other things has resorted to Homœopathy, which, as his ailment is a real one, and one not likely to get well without remedies, or by an influence exerted on the imagination, was, of course, inoperative.

*Remarks.*—There are several pathological conditions which may give rise to cephalalgia. Among these are (a) active determination of blood to the head ; (b) passive congestion ; (c) a dynamic condition analogous to neuralgia. In the latter case the affection seems sometimes located in the nerves of the scalp, and sometimes within the cranium. The present case is supposed to be of the neuralgic class. The remedy which we will make trial of, is as follows :—

℞ Sulph. Quiniae grs. iii.

three times daily.

This patient did not again present himself to report the effect of the prescription.

*March 3d.*—*Chronic Bronchitis.*—M. M., aet. 35, seaman. Three months ago had fever which was called congestive fever. Has never been well since. Had cough during the fever, which has continued ever since. It is a loud, violent cough, and expectoration is difficult. Complains of chills daily, which are followed by febrile movement, and sweating. Has lost flesh, countenance is pallid.

On examination of chest, resonance on percussion generally seems rather dull, but dullness is not well marked on either side. A sibilant rale is present at the summit of the right lung.

*Remarks.*—It is sometimes exceedingly difficult to decide after a a single examination of a case whether it be one of chronic bronchitis, or pulmo-tuberculosis. If the tubercular deposits are small, and pretty generally disseminated, the character of the affection may not be well declared by appreciable physical signs, and, on the other hand, chronic bronchitis may present all the rational or vital symptoms, local and general, of true Phthisis. We can only form an opinion, after repeated examinations, by carefully weighing every circumstance, and balancing all the symptoms. In view of the general phenomena of this case, I am disposed to think it is a case of tuberculosis. Although he has had Int. fever, it is probable that the daily paroxysms are to be regarded as hectic. The following is prescribed: ℞. Bals. Copaibae ℥ss; S. Morph. gr. i; P. Gum acaciae ʒij.; Aquae ℥iv.; F, Mistura. Teaspoonful to be taken four times daily. Liniment of Acetic acid and spirits Terebinth, (Stokes' liniment) to chest night and morning.

*March* 11*th*.—Patient reports that cough is somewhat relieved. Daily paroxysms of chills, febrile movement and sweating, continue.

*Remarks*.—Although the pulmonary symptoms appear to be improved, this does not remove the suspicions of tuberculosis, because the tubercles may be complicated with bronchitis, which the remedy may relieve, but without procuring permanent benefit. I am still disposed to think the paroxysms are hectic, but I will prescribe the Quinia. I have employed this remedy in cases of undoubted hectic, not only without injury, but occasionally with relief to this troublesome symptom of Phthisis. ℞. S. Quiniae, ʒi.; Elix. vitriol. ʒss.; Aquæ Destil, ℥ii. Teaspoonful every four hours.

*April* 2*d*.—Patient presented himself, and reported that the chills ceased shortly after taking the quinia, and that the pulmonary symptoms had entirely disappeared. He has gained flesh, and his aspect is greatly improved. He considers himself well, and able to labour.

*Remarks*.—The unfavourable opinion at first entertained of this case has been happily disproved by the result. It was a case of chronic bronchitis, associated with intermittent fever. We not unfrequently meet with similar cases, in which, if we venture upon a positive diagnosis at the outset, we shall often find ourselves deceived. The liability in early medical life is to err in regarding cases of incipient Phthisis as simply cases of bronchitis. The earnest wishes of the Practitioner warp his judgement; he flatters himself that nothing but bronchitis is present, because, for the sake of the patient, he hopes it is so. After several mistakes of this kind, he not only learns caution, but may be disposed to take too unfavourable a view of all cases of a doubtful character. Hence, he becomes liable to commit errors of an opposite character to those which previously befell his experience.

In all instances of successful treatment, it is well to consider how much of the effect is fairly due to the remedies employed. In this case the remedies have doubtless been efficient, but I am disposed to attribute considerable agency to the favourable influence of the season. Patients of this class during the cold weather are unfavourably situated to recover from pulmonary disease, and warm weather alone often does more than medicine. In the poorer classes of society, among those whose habits are reckless and dissipated, chronic bronchitis is especially apt to continue, and to assume the aspect of tubercular disease, frequently without ending in Phthisis, although greatly protracted by a constant succession of imprudences, and the want of proper medical treatment. This fact goes strongly to sustain the doctrine, that tuberculosis involves, in addition to local pulmonary causes of disease, a constitutional diathesis for its developement.

*Albuminaria.*—A boy aet. 16, has been ill for two or three weeks. Complains of pain in the loins, and over pubis ; some pain in micturition, urine high coloured, and occasionally appearing to contain blood. Conjoined with these symptoms are loss of appetite, and general debility. Previously to present illness was in good health.

Urine, on inspection, presents a smoky appearance. On being tested with nitric acid, and by heat, throws down a copious deposit of albumen.

*Remarks.*—The disease in this case is manifestly located in the kidneys. The affection is recent. There are, as yet, none of the secondary disorders arising from the peculiar lesion of the renal organs, which constitutes confirmed albuminaria, or *morbus Brightii.* The kidneys are probably in a state of congestion. The affection is not traceable to any cause. It has probably arisen from some exposure to cold, although the patient is not aware of any particular imprudence to which it may be assigned.

The treatment to be reccommended is cupping over the lumbar region ; confinement to the house ; mild saline laxatives ; and, afterward, tonics, such as quinia, and the citrate of iron. For the opportunity of presenting this case, I am indebted to my colleague, Dr. White, who will pursue the course of treatment mentioned.

This patient recovered in two weeks. A specimen of the urine, at the expiration of that time, tested before the class, exhibited a natural appearance, and was entirely devoid of albumen.

*Erythema Nodosum.*—Boy, aet. 8 years. Body and extremeties covered with red, circumscribed, hard blotches, countenance pallid.

*Remarks.*—This is a case of Erythema, and from the red patches being small, and accompanied with swelling, resembling bunches or nodosities, it belongs to the variety of this genus, called *Erythema nodosum.* It is a trivial affection in so far as the eruption is concerned. It obviously proceeds from disturbance of the general health. The pallid aspect of the child, denotes that the latter is at fault. He has suffered of late from headache, so that I have apprehended some latent disease of the encephalon. The aspect leads to the suspicion of the scrofulous cachexy. As costiveness is present, we will direct, first, a few grains of calomel, and subsequently, the liquor, iodid. Ferri.

This patient presented himself some time afterward. The erythema had entirely disappeared, and his aspect was much improved.

*Enlargement of Submaxillary glands.—Remarks.*—This patient is a child one year old. The submaxillary glands on the left side have suppurated, and on both sides the glands are enlarged. This affection is liable to be confounded with scrofula. It is a sympathetic affection in this case, caused by the irritation of teething. Whenever you meet with such swellings in children, you are to examine the gums. They do not denote scrofula when thus connected, unless they are associated with other indications of the scrofulous diathesis.

I have scarified the gums freely, and prescribed a laxative.

*Eczema Capitis.*—A boy aged about 13. Has been affected with eczema, located on the posterior part of the head, for four years.—Has tried a great number of local applications. The hair has never been cut closely, nor ablutions diligently pursued.

*Remarks.*—If the hair be kept closely cut, and the incrustations daily removed by means of warm fomentations, the disease will probably in time get well. We will direct this to be done, but it is very doubtful if it be faithfully practiced, or persevered in sufficiently long. It is difficult to make persons appreciate the importance of simple cleanliness ; or it imposes too much trouble. They prefer to try local applications, which, if cleanliness be not attended to, are seldom of any benefit. The patient complains of pruritus, which causes him to scratch the diseased part, and, in this way, the irritation is kept up. To alleviate this symptom, we will prescribe the Kreosote ointment.

*Psoriasis.*—A negro, 25 years of age, presents on back and shoulders a cutaneous eruption in rings and patches, from the size of a shilling to that of a dollar.

*Remarks.*—It is somewhat more difficult to diagnosticate cutaneous eruptions on a black than on a white skin. The first point is, if possible, to ascertain what are the elementary characters. This is not always practicable if the disease has existed for some time, as it has in the present case. We can however determine whether it is a *moist* or *dry* eruption. The patient states that this has been from the first dry. It does not, belong, then, to the class of vesicular eruptions, although it bears, at first view, a close resemblance to *Herpes circinatus.* It must belong either to the class of papulae, or squamae, and the appearances evidently locate it in the latter. There are, as you know, three genera of this order, viz : Pityriassis, Lepra, and Psoriasis. This evidently does not come within the first genus. The two latter, for all practical purposes, might be united into a single genus. The present case, however, belongs to Psoriasis, and, from its annular form, is called *Psoriasis Gyrata.* The ring is thus formed : first a scaly patch occurs which extends itself by the enlargement of its circumference, and while it is thus enlarging at its margin, the centre of the patch heals, which causes it to assume the form of a scaly ring.

The treatment advised in the Liquor arsen. pot. gtt., iv. 3 times daily, increased by one drop daily, up to ten drops.

*March 23d.—Chronic Bronchitis.*—J. B., seaman, has had Int. fever lately, which was cured by Quinia. It was followed by cough. Expectoration small in quantity, and viscid. Some dull pain in region of sternum. Cough is especially troublesome at night. Aspect denotes robust health ; no febrile movement ; appetite and digestive functions little, if at all impaired. Treatment, thus far, has consisted, simply, of stimulating liniment to chest, and a few grains of Dover's powder at night.

The physical signs are, good resonance on percussion over entire chest, yesterday feebleness of respiratory murmur was observed over the right side, which led to the supposition that the bronchitis might be confined to that side ; but today the murmur is feeble on both sides. No rales.

*Remarks.*—The case is one of subacute Bronchitis, which, with proper hygienic care, and some medication, will probably get well in a short time. The patient is apparently of temperate habits.

I shall prescribe a remedy in this case, which you will observe me generally to employ in similar cases. This is the Copaiva Emulsion, conjoined with a little sulphate of morphia. *In a large proportion of cases of chronic or subacute Bronchitis, the Copaiva appears to have a specific effect in removing the disease scarcely less than it exerts in urethritis.*

*March 26th.*—J. L., aged 5 years. Had Rubeola three weeks ago. The catarrhal, or bronchial symptons continue. The patient now presents high febrile excitement—hot skin, frequent pulse, &c. There is no evidence of embarassed respiration, except what would attend the increased activity of the circulation. The bowels are costive.

*Remarks.*—In all cases of local disease existing with sympathetic fever, we are to consider whether the local symptoms denote a degree of disease commensurate with the general disturbance of the economy. There is evidently bronchitis in this case, which is apt to remain after Rubeola ; but there are no local evidences that the functions of the lungs are seriously compromised. The respiratory acts are not much more frequent than natural, the alae nasi do not dilate, nor do the prolabia denote imperfect hæmatosis. As the bowels are costive, I am disposed to think that the present febrile movement is due, not so much to the bronchitis, as to the state of the alimentary canal. I will, accordingly, direct a cathartic of ol. Ricini.

*March 27th.*—Presented and reported much better. Febrile symptoms relieved by the operation of the oil. Cough &c. slight. Directed mixture of Syrup Ipecacuanhae and syrup Bals. Tolu., equal parts, a teaspoonful to be administered every six hours. Diet to be regulated, and other hygienic precautions attended to.

*Pulmo-tuberculosis.*—M. M., aged 35. Labourer. Contracted a cold a year ago. Has coughed more or less since, at some times more than at others. Been constantly loosing flesh and strength. Appetite is poor. Has never had pains in chest. Is easily put out of breath on exertion. Has never had chills. Expectorates most in the morning. Sweats every night. Physical signs :—Bronchial respiration on both sides, and obvious dullness on percussion on the left side. Pulse not accelerated, and rather small.

*Remarks.*—The physical signs in this case determine the diagnosis to be tuberculosis. There is not present that febrile movement, or accelerated pulse, (tuberculous fever) which frequently attends tubercular disease, and denotes that the disease is rapidly progressive. Probably the tubercular diathesis has been superinduced upon previously existing bronchitis. We will prescribe the copaiva mixture in this case.

*April 6th.*—Reported cough to be somewhat improved, and expectoration diminished, Bowels are very costive, and complains of having no appetite.

* *Remarks.*—In order to appreciate the influence of the copaiva in producing improvement in the cough and expectoration, we will omit this remedy, and prescribe the tinct. Aloes and Myrrh to obviate costiveness. It is not, however, always safe to trust reports of improvement in such cases. Patients with this disease are very apt to deceive themselves in this respect.

*April 12th.*—Reports that medicine produced moderately laxative effects. Cough has been greater for the past week, than for the previous week. Expectoration, continues about the same. Thinks he is a little stronger than at last report, and states that appetite is somewhat improved. Has not sweat since he took the last medicine.

*Remarks.*—From the general symptoms of this case, I conclude that the tubercular disease is not making rapid progress, and that the tuberculous cachexy is not strong. Nevertheless, he will finally succumb to the disease. I know of no better prescription than the copaiva mixture, and will accordingly resume it, omitting the morphia, in order to secure a laxative operation.

---

* Remarks which would have an unfavourable effect, are not,of course, made while the patient is before the class.

*Intercostal neuralgia—Masked Intermittent.—March 30th.*—M. H., aged 18, seaman. Complains of pain in the left chest, aggravated by each inspiration. Occasionally has pain in the head, and in loins. Complexion is pallid. Pulse not accelerated. Tongue furred. No derangement of digestive functions. Is costive. No tenderness of spine.

*Remarks.*—This is a case of intercostal neuralgia. The character of the pain causes it sometimes to be confounded with pleuritis, but an error of this kind is inexcusable, since the concomitant symptoms of an inflammatory affection are absent. The mistake, nevertheless, is sometimes made, and it is always unfortunate, because depletion, under such circumstances, generally injures the patient. We will today simply prescribe a cathartic, and the application of a strong liniment to chest.

*April 5th.*—Reports little if at all better. Tongue is covered with a thin very white fur, and he complains of sweating every night. Has never had Remittent nor Intermittent fever.

*Remarks.*—I am now satisfied that this is a case of latent, or rather masked Intermittent, and I predict with great confidence, that in taking Quinia for a few days, he will be well. I prescribed the sesqui-oxide, of Iron since he last presented himself, being guided chiefly by his pallid complexion, before arriving at any present diagnosis. The opinion that it is latent Intermittent, is based upon the peculiar frosted tongue, and the nightly sweating, conjoined with the fact that during the last season, he was in localities where miasmata abounded, although he has never had Remit. or Intermit. fever. I suppose there is an abortive attempt at a Quotidian paroxysm, although he has no chills, and is not sensible that febrile movement precedes the sweating.

℞. Quinia, grs. iii., four times daily. Sesqui-oxide of Iron to be continued.

*April 10th.*—Reports better. Pain is diminished. No sweating at night. Feels stronger. Same treatment continued.

*April 15th.*—Reports quite well. Feels slight intercostal pain occasionally, but considers himself sufficiently well to go to work. Aspect is greatly improved.

*April 5th.—Pulmo-Tuberculosis.*—G. L. Dates his present illness from Nov. 1st, 1846. Previously was not healthy for several years, but was not aware of any ailment except debility.

Was first taken with pain in loins, which was severe, and continued for five or six weeks. Then, the pain shifted upward under the scapula. Then, commenced cough, attended with fever in the afternoon, and sweating at night. Cough was at first dry. Pain has continued more or less ever since, shifting its locations, occurring first in the left, and afterwards in the right chest. It is sharp like a stitch. Sweating at night has now ceased, but thinks that the fever continues. Had chills when he had paroxysms of fever and sweating. Has expectorated blood, at times profusely. Has lost flesh and strength constantly. Is easily put out of breath on exertion.

Physical signs: very tender over the right chest, so as not to bear even slight percussion, nor the pressure of the stethoscope.—The same excessive tenderness has previously existed in the left side. Dullness, approaching to flatness, over the left chest. Respiration in some portions absent, in others bronchial.

*Remarks.*—After this general view of the phenomena, it is, I trust, hardly necessary to say, that the diagnosis is, unequivocally, Pulmo-tuberculosis. The pain and tenderness in chest denote pleuritis, such as generally occurs in connection with the tubercular deposit, and is a conservative measure, furnishing a security against rupture or perforation of the pleura over abscesses an accident which occasionally occurs, and hurries the patient to a speedy dissolution. No remedies will afford this patient any permanent benefit. We will prescribe S. Morphia in the Syrup bals. Tolu., to alleviate the cough.

*April 7th—Gastralgia.*—S. W., aged about 30, joiner. Has been an invalid for a year, previously having enjoyed excellent health.—Complained first, of a hard cough, which continued for three weeks. Supposed he had contracted a severe cold. Cough ceased, and he has had it only occasionally since, when, as he has supposed, he has contracted cold. Shortly after cessation of cough, began to experience pains in the back, between the scapulae, and in the Epigastrium. These pains gradually increased for two or three months, and, since then, have continued about stationary. The character of the pain is dull, not acute. He calls it an aching pain. He is conscious of the pain most of the time during the day, but is free from it during the night. It is considerably harder at some times than at others. It is most severe in the Epigastrium.

He has lost some, but not much flesh. His weight has diminished only three pounds since last June. His strength has constantly diminished, but he is by no means feeble. Until within two months his appetite has been tolerably good, but, since then, has been poor. Is not aware that the ingestion of food ever increases the pain, or produces any inconveniences. Tongue is slightly furred. Bowels are generally regular. Extremeties are disposed to be cold. Pulse natural. A full respiration produces some increase of pain, more at some time than at others. Mind is depressed and desponding. There is tenderness over epigastrium frequently, but not uniformly. If he elevates the abdomen, and suddenly allows it to fall, it occasions pain. Sudden jolts, also, produce pain at Epigastrium. Has not laboured for the past year.

*Remarks.*—This patient was examined by me last Nov. I then explored the chest and abdomen, and tested the urine, without discovering any pathological phenomena worthy of note. The treatment heretofore pursued has been various botanic remedies, and by Dr. Wakely, of Clarence, a Practitioner of great respectability, the spine was kept sore for two months with the ointment of tartrate of antimony. He has had irritating plasters over the epigastrium, been vesicated over that region several times, and, also, between the shoulders. I prescribed the sulphate of Iron last Nov., which he took for a month. Has taken large quantities of sarsaparilla. As regards the diagnosis, there is either some organic affection of stomach, or adjoining vsicera ; or it is a neurotic affection. I am disposed to take the latter view. It is, then, nosologically, a case of gastralgia. There are not present the local symptoms which we should expect to find associated with an organic lesion of the stomach or neighboring parts, productive of such long continued pain. The general condition of the economy is not that which denotes inflammation, but, on the contrary, that which is characteristic of a neuropathic affection. There is an obscure tenderness on pressure over the dorsal vertebrae, and I am disposed to think the pain proceeds from a morbid condition of the nerves at their connection with the spinal cord, or of the cord itself. Had not counter irritation been so efficiently pursued by Dr. Wakely, I should strongly reccommend it ; as it is, I will prescribe the subnitrate of Bismuth, a remedy which is supposed to have an excellent effect in gastralgia. We will commence with grs. iii, three times daily, and gradually increase the quantity. I suspect hypochondriasis is involved in this case. The patient is very anxious about his health, and, perhaps, imagines he is much more afflicted than he is. It would be well for him to engage in occupation as much as practicable, to divert his mind from his morbid sensations.

*April 14th.*—Reports the same. Bismuth to be increased to grs. v., three times daily.

